# SNP Discovery by Illumina-Based Transcriptome Sequencing of the Olive and the Genetic Characterization of Turkish Olive Genotypes Revealed by AFLP, SSR and SNP Markers

**DOI:** 10.1371/journal.pone.0073674

**Published:** 2013-09-13

**Authors:** Hilal Betul Kaya, Oznur Cetin, Hulya Kaya, Mustafa Sahin, Filiz Sefer, Abdullah Kahraman, Bahattin Tanyolac

**Affiliations:** 1 Department of Bioengineering, Ege University, Izmir, Turkey; 2 Olive Research Station, Izmir, Turkey; 3 Department of Field Crops, Harran University, S. Urfa, Turkey; Harbin Institute of Technology, China

## Abstract

**Background:**

The olive tree (*Olea europaea* L.) is a diploid (2n = 2x = 46) outcrossing species mainly grown in the Mediterranean area, where it is the most important oil-producing crop. Because of its economic, cultural and ecological importance, various DNA markers have been used in the olive to characterize and elucidate homonyms, synonyms and unknown accessions. However, a comprehensive characterization and a full sequence of its transcriptome are unavailable, leading to the importance of an efficient large-scale single nucleotide polymorphism (SNP) discovery in olive. The objectives of this study were (1) to discover olive SNPs using next-generation sequencing and to identify SNP primers for cultivar identification and (2) to characterize 96 olive genotypes originating from different regions of Turkey.

**Methodology/Principal Findings:**

Next-generation sequencing technology was used with five distinct olive genotypes and generated cDNA, producing 126,542,413 reads using an Illumina Genome Analyzer IIx. Following quality and size trimming, the high-quality reads were assembled into 22,052 contigs with an average length of 1,321 bases and 45 singletons. The SNPs were filtered and 2,987 high-quality putative SNP primers were identified. The assembled sequences and singletons were subjected to BLAST similarity searches and annotated with a Gene Ontology identifier. To identify the 96 olive genotypes, these SNP primers were applied to the genotypes in combination with ***amplified fragment length polymorphism*** (AFLP) and ***simple sequence repeats*** (SSR) markers.

**Conclusions/Significance:**

This study marks the highest number of SNP markers discovered to date from olive genotypes using transcriptome sequencing. The developed SNP markers will provide a useful source for molecular genetic studies, such as genetic diversity and characterization, high density quantitative trait locus (QTL) analysis, association mapping and map-based gene cloning in the olive. High levels of genetic variation among Turkish olive genotypes revealed by SNPs, AFLPs and SSRs allowed us to characterize the Turkish olive genotype.

## Introduction

The olive tree (*Olea europaea* L. *subsp. europaea var. europaea, Oleaceae*) is one of the most ancient and important Mediterranean long-lived fruit species [1]. It is a diploid (2n = 2x = 46) outcrossing species mainly grown in the Mediterranean basin with a very wide genetic patrimony [2]. This wide genetic patrimony is represented by more than 1200 cultivars [3]. Olive oil and table olives are very important components in the Mediterranean diet [4]. Several studies have emphasized the beneficial effects of table olives [4] and olive oil on human health [5].The leading olive-producing countries of the world are Spain, Italy, Greece and Morocco. According to statistics provided by the Food and Agriculture Organization (FAO), Turkey ranks as the fifth largest olive producer in the world, with production hovering approximately 1.415 million tons of fruit in 2010 [6].

The sequencing and analysis of transcriptomes has been considered an efficient approach for gene expression profiling, alternative splicing, SNP discovery, mapping and quantification of transcriptomes in plants, especially in species without a reference genome sequence [7], [8]. The Sanger sequencing of ESTs used to be the most common approach for SNP discovery to obtain the expressed sequence tags (ESTs) information. Over the past 10 years, the sequencing of ESTs using traditional techniques were used in several important species [9]. However, Sanger sequencing requires expensive and time-consuming approaches, including cDNA library construction and the cloning of DNA fragments [10]. Alternatively, a transcriptome analysis based on next-generation sequencing (NGS) is more attractive in identifying a transcriptome sequence dataset for marker development and gene discovery due to its lower cost per base pair of DNA, short time requirement and lack of a subcloning process [11]. Next-generation transcriptome sequencing has created transcriptome databases in various plants without a sequenced genome, including chickpea [12], wheat [13], *Eucalyptus pilularis* [14], carrot [15], mangroves [16], strawberry [17] and chestnut [18]. Additionally, the discovery of SNP markers using NGS technologies permits the identification of thousands of markers from entire genomes or from cDNA [19], which can be used for genetic diversity analyses [20], association mapping [21], [22], linkage mapping [23] and marker-assisted selection [24] studies.

Various platforms utilizing NGS, such as the Roche 454 Genome Sequencer, the Illumina Genome Analyzer and the Life Technologies SOLiD System, can produce massive sequence outputs, making high-throughput DNA marker discovery feasible and cost-effective [25], [26]. There are various advantages and limitations among the various NGS platforms, which vary in terms of sensitivity, accuracy, reproducibility and throughput. Among these platforms, Illumina sequencing technology, which generates large-scale reads (75–150 bp) at low costs with very high sequencing coverage, has been especially useful for de novo transcriptome studies [25]–[27].

A large number of accessions are currently available in olive-producing countries, raising several problems for germplasm management and preservation [28]. The evaluation and identification of olive genetic resources is therefore crucial, especially estimating the genetic variation in the existing germplasm, particularly due to the high occurrence of mislabeling, synonyms and homonyms in the olive.

Genetic identification is the first key step in breeding programs, and molecular markers are valuable tools for identifying and characterizing diverse genotypes [29]. Currently, with the large array of DNA molecular marker types available, DNA markers provide useful information in theoretical and applied research fields for olive breeding, such as the determination of genetic diversity, genetic relationships [30] and population structures among cultivated species and their wild relatives [31], [32]; the characterization of large olive germplasms [32]; and the traceability of olive oil to its cultivars [33]–[35]. A wide variety of polymerase chain reaction (PCR)-based molecular markers, such as AFLP [30], SSR [36]–[38], inter simple sequence repeat (ISSR) [39], [40], diversity arrays technology (DART) [32] and sequence-related amplified polymorphism (SRAP) [36] are used for characterization in the olive. However, the frequency of these markers in the genome is very low compared to that of SNP markers. Due to the lack of sequence information and the cost of the sequencing technique, there are a limited number of SNP markers used today. Because the olive genome has not yet been sequenced, this technique has not been widely applied. Although some SNPs have been developed from the olive genome, their number is limited [41]–[43] and they are reproducible for mapping studies [42] and cultivar identification [32].

In the present study, we utilized Illumina Genome Analyzer IIx (GAIIx) sequencing technology to perform *de novo* transcriptome sequencing of the olive and to develop EST-derived SNP markers. The SNP markers generated in this study can be used to characterize olive genotypes, to facilitate linkage map construction, to perform association mapping and to aid in marker-assisted selection. To date, molecular marker systems have been applied in Turkish olive varieties to examine the genetic diversity and differentiation among olive cultivars [36], [44]. The present study is the first to report the large-scale discovery of SNPs in the olive genome and the use of these SNPs in the molecular characterization of 96 olive genotypes from Turkish Olive GenBank Resources to not only determine the nature and extent of the genetic diversity in the olive genotypes but also to characterize the genetic structure of each genotype and to investigate the genetic relationships among olive genotypes.

## Materials and Methods

### Plant Material

**Figure 1:**
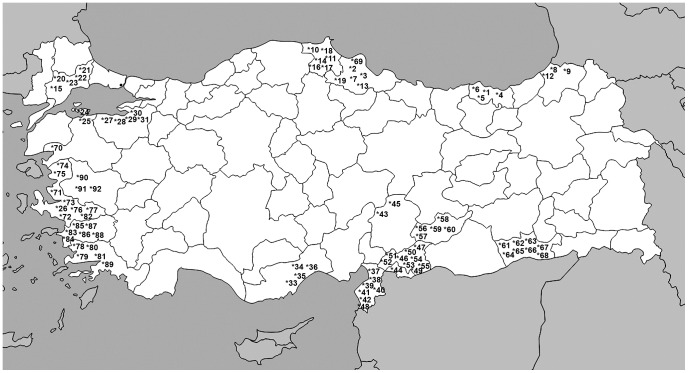
Map of Turkey indicating the location of the olive tree genotypes used in the study. See Table 1 for the code numbers.

A total of 96 olive genotypes (Table 1) were used in this study: 91 of the most important commercial olive cultivars together and 5 unknown genotypes, all grown in Turkey (Figure 1). For DNA isolation, the young leaves of the 96 olive genotypes were collected from the Turkish Olive GenBank Resources in Izmir-Turkey. The common name, origin, and end-use of all the genotypes are given in Table 1.

**Table 1:** 96 olive genotypes used in the study.

Code Number	Genotype	Location	Region	Use	Code Number	Genotype	Location	Region	Use
**1**	Trabzon Yağlık	Trabzon	Black Sea	Both use	49	Hamza Çelebi	Nizip	Southeastern	Both use
**2**	Samsun Yağlık	Samsun	Black Sea	Both use	50	Yuvarlak Halhalı	Nizip	Southeastern	Table
**3**	Görvele	Samsun	Black Sea	Oil	51	Kalem Bezi	Nizip	Southeastern	Oil
**4**	Marantelli 1	Trabzon	Black Sea	Table	52	Yağlık Çelebi	Nizip	Southeastern	Both use
**5**	Marantelli 2	Trabzon	Black Sea	Table	53	Yün Çelebi	Nizip	Southeastern	Table
**6**	Patos	Trabzon	Black Sea	Both use	54	Eğri Burun	Nizip	Southeastern	Table
**7**	Kırmızı tuzlamalık	Samsun	Black Sea	Table	55	Tesbih Çelebi	Nizip	Southeastern	Oil
**8**	Butko	Artvin	Black Sea	Both use	56	Eğri Burun	Tatayn	Southeastern	Both use
**9**	Otur	Artvin	Black Sea	Both use	57	Yuvarlak Çelebi	Tatayn	Southeastern	Table
**10**	Ağaç No 5	Sinop	Black Sea	Table	58	Hırhalı Çelebi	Tatayn	Southeastern	Table
**11**	Ağaç No 2	Sinop	Black Sea	Both use	59	İri Yuvarlak	Tatayn	Southeastern	Table
**12**	Satı	Artvin	Black Sea	Both use	60	Yağ Çelebi	Tatayn	Southeastern	Both use
**13**	Ufak tuzlamalık	Samsun	Black Sea	Table	61	Zoncuk	Derik	Southeastern	Table
**14**	Ağaç No 4	Sinop	Black Sea	Both use	62	Halhalı 1	Derik	Southeastern	Both use
**15**	Siyah Salamuralık	Tekirdağ	Marmara	Both use	63	Halhalı 2	Derik	Southeastern	Both use
**16**	Ağaç No 6	Sinop	Black Sea	Both use	64	Halhalı 3	Derik	Southeastern	Both use
**17**	Ağaç No 7	Sinop	Black Sea	Both use	65	Hursuki	Derik	Southeastern	Oil
**18**	Ağaç No 1	Sinop	Black Sea	Both use	66	Belluti	Derik	Southeastern	Both use
**19**	Samsun salamuralık	Samsun	Black Sea	Table	67	Melkabazı	Derik	Southeastern	Table
**20**	Beyaz Yağlık 1	Tekirdağ	Marmara	Both use	68	Mavı	Derik	Southeastern	Both use
**21**	Beyaz Yağlık 2	Tekirdağ	Marmara	Both use	69	Samsun Tuzlamalık	Samsun	Black Sea	Table
**22**	Çizmelik	Tekirdağ	Marmara	Table	70	Ayvalık Yağlık	Ayvalık	Marmara	Both use
**23**	Eşek Zeytini	Tekirdağ	Marmara	Both use	71	Hurma kabaca	İzmir	Aegean	Both use
**24**	Erdek Yağlık	Erdek	Marmara	Oil	72	Hurma Kaba	İzmir	Aegean	Both use
**25**	Edincik	Edincik	Marmara	Table	73	Erkence	İzmir	Aegean	Oil
**26**	Eşek Zeytini	Ödemiş	Aegean	Table	74	Çilli	İzmir	Aegean	Table
**27**	Gemlik	İznik	Marmara	Both use	75	İzmir Sofralık	İzmir	Aegean	Table
**28**	Su Zeytini	İznik	Marmara	Table	76	Çakır	İzmir	Aegean	Oil
**29**	Şam	İznik	Marmara	Table	77	Memeli	İzmir	Aegean	Both use
**30**	Samanlı	İznik	Marmara	Table	78	Dilmit	Bodrum	Aegean	Oil
**31**	Çelebi	İznik	Marmara	Table	79	Girit Zeytini	Bodrum	Aegean	Oil
**32**	undefined	–	–	–	80	Tavşan Yüreği	Milas	Aegean	Table
**33**	Büyük Topak Ulak	Tarsus	Mediterranean	Table	81	Ak Zeytin	Milas	Aegean	Both use
**34**	Sarı Ulak	Tarsus	Mediterranean	Table	82	Çekişte	Ödemiş	Aegean	Both use
**35**	Küçük Topak Ulak	Tarsus	Mediterranean	Oil	83	Kara Yaprak	Kuşadası	Aegean	Oil
**36**	Çelebi	Silifke	Mediterranean	Both use	84	Yağ Zeytini	Kuşadası	Aegean	Both use
**37**	Halhalı	Hatay	Mediterranean	Oil	85	Yerli Yağlık	Kuşadası	Aegean	Both use
**38**	Sarı Habeşi	Hatay	Mediterranean	Oil	86	Aşı Yeli	Aydın	Aegean	Both use
**39**	Saurani	Hatay	Mediterranean	Both use	87	Taş Arası	Aydın	Aegean	Oil
**40**	Sayfı	Hatay	Mediterranean	Oil	88	Taş Arası	Kuşadası	Aegean	Oil
**41**	Karamani	Hatay	Mediterranean	Both use	89	Memecik	Milas	Aegean	Both use
**42**	Elmacık	Hatay	Mediterranean	Table	90	Domat	Akhisar	Aegean	Table
**43**	Yağlık Sarı Zeytin	K.Maraş	Mediterranean	Both use	91	Kiraz	Akhisar	Aegean	Both use
**44**	Kilis Yağlık	Kilis	Southeastern	Oil	92	Uslu	Akhisar	Aegean	Table
**45**	Ağaç No 7	K.Maraş	Mediterranean	Oil	93	undefined	–	–	–
**46**	Nizip Yağlık	Nizip	Southeastern	Oil	94	undefined	–	–	–
**47**	Kan Çelebi	Nizip	Southeastern	Table	95	undefined	–	–	–
**48**	Halhalı Çelebi	Hatay	Mediterranean	Table	96	undefined	–	–	–

### Total RNA Extraction, cDNA Library Construction and Transcriptome Sequencing

**Figure 2:**
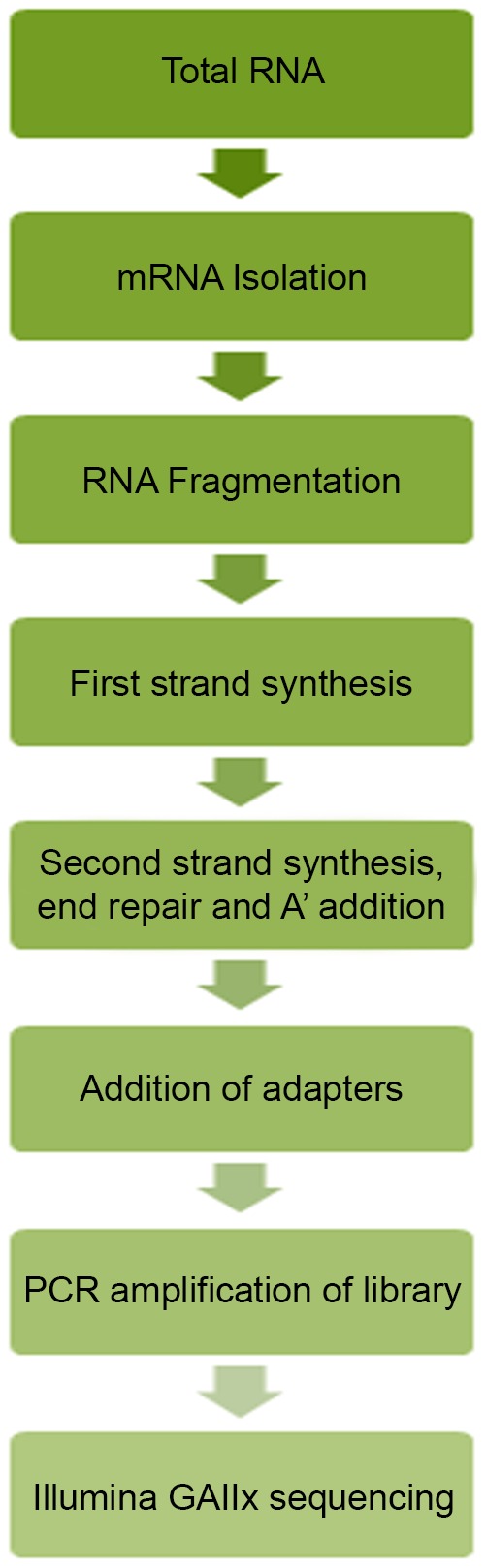
Overview of high-throughput RNA-seq library preparation. mRNA is isolated from total RNA and fragmented. The mRNA is used to make first and second strands of cDNA and this double stranded cDNA molecules are subsequently synthesized, end-repaired and adenylated. Illumina adaptors are ligated to the processed double-stranded DNA and size selected. Finally, the ligated samples are then enriched by amplification using adapter specific primers and purified for sequencing.

The SNP analysis used the RNA samples of five olive genotypes (Siyah Salamuralık, Yun Celebi, Yuvarlak Celebi, Hirhali Celebi and Halhali 3) that originated from different locations in Turkey. Total RNA was extracted using the RNeasy Plant Mini Kit (QIAGEN, CA, USA, Cat. Number: 74903). The RNA was quantified using Qubit (Invitrogen Inc. USA) and its quality was checked by running with a 0.8% agarose gel under denaturing conditions. The poly (A) mRNA was purified from the total RNA using the Oligotex mRNA Midi Prep Kit (QIAGEN, Cat Number: 70022) followed by repurification using the mRNA-Seq-8 Sample Prep Kit (Illumina Inc. San Diego, USA, Cat. No: # RS-100-0801). The poly-A containing mRNA was purified from 2 µg total RNA using oligo(dT) magnetic beads and fragmented into 200–500 bp pieces using divalent cations at 94°C for 5 min. The cleaved RNA fragments were copied into first strand cDNA using SuperScript II reverse transcriptase (Life Technologies, Inc.). After the second strand cDNA synthesis, the fragments were end-repaired and a-tailed and the indexed adapters were ligated. The products were purified and enriched by PCR to create the final cDNA library. These pooled libraries were sequenced at the DNA Link, Inc. in Seoul, South Korea using an Illumina GAIIx (Illumina Inc., San Diego, CA, USA). The workflow is shown in Figure 2.

### Analysis of Illumina Transcriptome Sequencing and SNP Discovery

**Figure 3:**
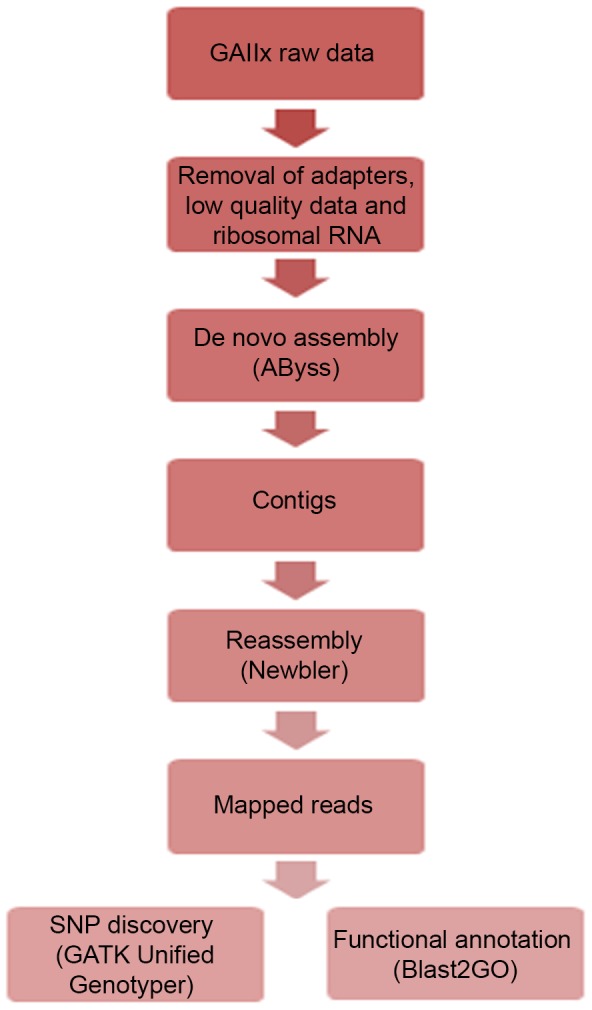
Workflow for *De novo* transcriptome assembly and analysis.

The raw sequencing data were transformed by base calling into sequence data (i.e., raw data or raw reads) stored in FASTQ format. Next, we used cutadapt (https://code.google.com/p/cutadapt/) [45] to remove any reads that were contaminated with an Illumina adapter. Then, the low-quality score regions and reads shorter than 70 bp were removed using our in-house script. In addition, a comprehensive ribosomal RNA database, the SILVA DATABASE (DB) [46], containing regularly updated, high-quality sequences of eukaryotic rRNAs was incorporated into the cleaning pipeline to remove ribosomal RNA sequences. Reads that mapped to SILVA DB sequences were assumed to be ribosomal RNA and were removed. The resulting non-mapped reads were then considered to be mRNA. These cleaned mRNA reads were assembled using ABySS tools [47]. The assembled contigs were reassembled using the de novo assembly tool Newbler version 2.3 (GS de novo assembler, Roche Applied Sciences). The cleaned mRNA reads (reads that did not map to SILVA DB sequences) were then mapped to Newbler’s output contigs, which were used as reference sequences. To validate these results, we used the Genome Analysis Toolkit (GATK) Unified Genotyper Algorithm [48] to independently identify the SNPs. Afterwards, the discovered SNPs were called coding SNPs (cSNPs) since they were generated originally from RNA transcripts. For gene annotation, we used the Blast2GO program [49] to obtain the Gene Ontology (GO) terms describing the biological process, molecular function and cellular components of the query sequences of the unigenes. A simplified workflow of the transcriptome assembly and bioinformatic analysis is shown in Figure 3.

### DNA Isolation

The young leaves of each genotype were harvested and stored at −80°C. The total genomic DNA was extracted by the CTAB method of Doyle and Doyle [50]. The isolated DNA was dissolved in TE buffer and incubated with RNase A (Fermentas) and Proteinase K (Fermentas) at 37°C for 1 h to remove RNAs and proteins. All DNA samples isolated from 96 olive genotypes were subjected to AFLP, SSR and SNP assays.

### AFLP Marker Genotyping

The AFLP procedure was performed according to Vos et al. [51] using a LI-COR (LI-COR Bioscience Lincoln, NE-USA) AFLP Kit (catalog number: 830-06197 AFLP 2-DYE Selective Amplification Kit). Specifically, the DNA samples were digested with the endonucleases *Eco*RI and *Mse*I and ligated to the appropriate double-stranded adapters. Two amplification steps followed: (1) a pre-selective amplification with primers carrying one selective nucleotide (*Mse*I-A, *EcoR*I-C) and (2) a selective amplification with primers carrying three bp extensions (*Mse*I+3/*Eco*RI+3), thereby further reducing the number of fragments. A total of twenty six primer combinations of *EcoR*I and *Mse*I with three nucleotides extension at 3′ ends were used. All of the PCR amplifications were conducted on a PTC 100 thermal cycler (MJ Research, Waltham, MA). The PCR products were run on 8% denaturing polyacrylamide gels. The PCR products were fractionated on a LI-COR 4300S DNA analyzer equipped with two infrared lasers with the ability to read at two wavelengths: 700 and 800 nm. Only bright, clearly distinguishable bands between 50 and 700 bp were recorded for analysis.

### SSR Marker Genotyping

A total of 14 microsatellite loci (DCA7, DCA11, DCA13, DCA15 and DCA18 [52]; and GAPU-71A, GAPU-71B, GAPU-82, GAPU-89, GAPU-90, GAPU-92, GAPU-101, GAPU-103A, and GAPU-108 [53]) were used to genotype the samples. The amplifications were performed in 20 µl reactions containing 0.25 U GoTaq Flexi DNA Polymerase (Promega, Madison, WI, USA), 1X Promega colorless GoTaq Flexi Buffer, 20 mM MgCl_2_, 0.2 mM each dNTP, 0.4 µM reverse primer, 0.1 µM extended forward primer, 0.4 µM labeled M13 primer (Eurofins MWG Operon, Huntsville, AL) and 100 ng/µl template DNA. The Maccaferri et al. [54] thermal cycling protocol was used for all of the primer sets, and the SSR profiles of the genotypes were obtained using the automated LI-COR 4300S DNA analyzer (LI-COR, Lincoln, NE, USA). For analysis on the LI-COR 4300S analyzer, the PCR products were added in a ratio of 1∶50 to the gel loading buffer (98% formamide, 10 mM EDTA and 0.5% bromophenol blue), heated for 3 min at 95°C, chilled quickly on ice and separated on a 6% acrylamide gel. We determined the size of alleles with the IRDye 50–700 bp fragment size ladder (LI-COR, USA).

### SNP Marker Genotyping

In the study, cSNP analyses were carried out using 140 of 2986 primers, which were developed in the transcriptome sequencing; amplification occurred in 49 of these primers. cSNP primers sequences that revealed amplification and annealing temperatures obtained from gradient PCR are given in Table 2. PCR amplifications were performed using the GoTaq® Flexi DNA Polymerase (Promega, Madison, WI, USA). Polymerase chain reactions were performed following the compositions described by Hakim et al. [43]. Amplified products were separated on 2% agarose gels in 1X Tris-acetate-EDTA buffer. Conditions of the PCR amplification were as follows: 95°C (4 min), 35 cycles at 95°C (30 sec), the appropriate annealing temperature (30 sec), and 72°C (1 min) and a final extension at 72°C for 6 min.

**Table 2:** Primer sequences, annealing temperatures, number of polymorphic bands and PIC values for 49 SNP primers used in the study.

SNPPrimer	Forward Primer Sequence	Reverse Primer Sequence	PrimerAnnealing	No. ofpolymorphicbands	PIC
SNP0002	ACTGTTACTCAAAGCATGCCTATT	TCAGATGAGGAAGCTGCAG	57°C	4	0.555
SNP0006	TGTGCTCATCTTGCCACG	TGGGATGCTAAGAACATGATG	52°C	2	0.552
SNP0008	TACTCAGCMACTAAATCATTCATGT	TTCAACTTTTATTGGTAACTCCACA	57°C	2	0.099
SNP0009	ACTAAATCATTCATGTTGCTCTCA	ATCAATCCAGGAGATGTTTAGG	59°C	1	0.01
SNP0010	ACTAAATCATTCATGTTGCTCTCA	ATCAATCCAGGAGATGTTTAGG	51°C	1	0.01
SNP0012	ATTGATGTGGAGTTACCAATAAAAG	AAAGGAGGTTGACGAGCAG	46°C	2	0.047
SNP0013	ATTGATGTGGAGTTACCAATAAAAG	AAAGGAGGTTGACGAGCAG	46°C	4	0.065
SNP0014	CGTCATACTCCTCATCAGCAG	AAGGCTTTCCTGCATTGG	46°C	4	0.448
SNP0015	TACTCAGCMACTAAATCATTCATGT	TTCAACTTTTATTGGTAACTCCACA	60°C	2	0.479
SNP0016	ACTAAATCATTCATGTTGCTCTCA	ATCAATCCAGGAGATGTTTAGG	62°C	1	0.083
SNP0017	ACTAAATCATTCATGTTGCTCTCA	ATCAATCCAGGAGATGTTTAGG	48°C	1	0.01
SNP0018	ATCTCRTCCATRCCTTCTCC	ATGGCTTCAACTTTTATTGGTAAC	48°C	2	0.427
SNP0019	ACAAACAGTTGACTTGACATTATTTG	ATCCTCGTCATGGTCGTTATT	48°C	1	0.116
SNP0020	AAGAGTTTTTGTTCTGGACATTCA	AGCTTACTCAACAGATGTGGGA	48°C	8	0.682
SNP0021	ATCATGTTCTTTGAATCCCACA	ATCTCCGCAAACTTGCTGT	59°C	1	0.813
SNP0022	ATCATGTTCTTTGAATCCCACA	ATCTCCGCAAACTTGCTGT	59°C	1	0.667
SNP0023	TTCTTTGAATCCCACATCTGTT	ATCTCCGCAAACTTGCTGT	46°C	1	0.271
SNP0024	AAAAGGTTTTGGGTTTGTCAA	TTTTCAGTTCCTGCTCTCTCTC	59°C	1	0.125
SNP0025	TGAAAAGTCGATTTGAGCAGA	ATCAATGCTGTCATCCAAATTT	59°C	1	0.083
SNP0026	TTGAGCAGAGTGCAAAGGA	GTTTGTTGTCATCAATGCTGTC	59°C	1	0.177
SNP0027	AAACTCAAGGAACTGTTTTCGG	TTCAGGAGCTGTGAATGCA	59°C	1	0.188
SNP0029	TTGAGCAGAGTGCAAAGGA	GTTTGTTGTCATCAATGCTGTC	46°C	4	0.602
SNP0030	AAACTCAAGGAACTGTTTTCGG	TTCAGGAGCTGTGAATGCA	46°C	1	0.01
SNP0035	AAACTCAAGGAACTGTTTTCGG	TTCAGGAGCTGTGAATGCA	46°C	2	0.021
SNP0038	CATGTTTTAACTTTTCCATTTGAC	AAATTGGTTCACTTTGGATCG	46°C	1	0.271
SNP0039	TCGGAGCCACCAACCCAG	CACTTCCGTGGATGACATTC	46°C	1	0.01
SNP0040	TTCATCAATGGATCTACGAGTG	TTCATRGAACAAAGTTCAAGTAACTC	46°C	1	0.313
SNP0042	TTGGATCCATGATTATATGTGC	ATCATATTCAAACAAAAACGCTC	46°C	1	0.073
SNP0043	TTGGATCCATGATTATATGTGC	ATCATATTCAAACAAAAACGCTC	46°C	2	0.521
SNP0044	TCGGAGCCACCAACCCAG	CACTTCCGTGGATGACATTC	46°C	1	0.042
SNP0047	AAAATGAGTGCAGAGCCC	ATTTTTACCATCACATCCTGTG	45°C	3	0.076
SNP0048	AACAGTACCATTGACACCACG	TCATTTTGCCAATATCATACACC	48°C	1	0.198
SNP0054	ATCCTTCTCTTGGACGTTGC	AAAACTTGAGACTTCTTGGTTGG	46°C	1	0.052
SNP0059	AAGTATTCTGAGTGGAGAGGGTG	AAAACTTGAGACTTCTTGGTTGG	66°C	1	0.26
SNP0062	ATCCTTCTCTTGGACGTTGC	AAAACTTGAGACTTCTTGGTTGG	62°C	1	0.146
SNP0075	AATATGACTTTTGCAAATATTCGG	TTATTAATCTTACCAAATCTCGTAGCA	62°C	2	0.229
SNP0080	AAAATGCAACGGAAAGCA	CTCCTGAACTTCCKGAACC	62°C	1	0.26
SNP0081	AACRAGTGATAACCAGTCCTTTTC	TTTATGGACTTCCAAATGAGACA	60°C	1	0.26
SNP0083	ACTGTGAACTGCAACRAGTGA	TCTGTCTTTATGGACTTCCAAAT	54°C	4	0.927
SNP0084	AACRAGTGATAACCAGTCCTTTTC	AAATGAGACRTGGGAAGTCAA	62°C	9	0.631
SNP0088	TTACTGGTGAAATTGGTGCTC	AATACTCTCAGTAACCGATCCAATT	60°C	1	0.927
SNP0089	TGGTGAAATTGGTGCTCAA	TCTTCTCCTTGATTGCCTTCT	54°C	1	0.99
SNP0098	ATATGGCAATGAGAACATGGA	TCAACAAGGGGTTTTGCA	62°C	1	0.24
SNP0118	ATCGCCTGGCAACGATTT	GGAACTGCATGTGGCAAA	62°C	1	0.448
SNP0123	ATCGCCTGGCAACGATTT	GGAACTGCATGTGGCAAA	60°C	2	0.302
SNP0127	TTTCAAAACCCTTACTGCCC	ATTAGCCCAAATGTTCCTTCC	54°C	1	0.24
SNP0131	AGAAAACTTTCCCTCTTCTTTCTC	AATTGGTGAGATTCAAGGTCTTT	62°C	1	0.24
SNP0132	AGAAAACTTTCCCTCTTCTTTCTC	AATTGGTGAGATTCAAGGTCTTT	62°C	1	0.729

### Marker Data Analysis

#### Polymorphism and discrimination power

The polymorphic AFLP fragments were scored as binary data, with the presence of bands denoted as ‘1’ and their absence as ‘0’, based on the AFLP pattern amplified by each primer combination. The codominant SSR and cSNP data were transformed into dominant data by treating each polymorphic band as a single locus coded by 1 (presence) or 0 (absence) and then combining these data with the AFLP data to create the dataset. The discrimination power of the combined marker was evaluated by the polymorphism information content (PIC) using the formula
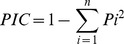
where *n* is the total number of alleles detected for a given marker locus and *Pi* is the frequency of the *i^th^* allele in the set of genotypes investigated [55], [56].

### Hierarchical Cluster Analysis

The combined data were exported into a spreadsheet and formatted for the NTSYSpc (v. 2.1) cluster analysis software (Exeter Software Co., New York). Jaccard’s coefficient was used to calculate the pairwise genetic similarities. A cluster analysis was performed on the genetic similarity matrix using the unweighted pair group method using arithmetic means (UPGMA) algorithm provided in the software package NTSYSpc. Principal coordinate analysis (PCA) was performed based on the genetic similarity matrix using the D center and Eigen functions of NTSYSpc [57].

### Bayesian Model Based Cluster Analysis

The model-based program STRUCTURE v 2.3.4 [58] was used to infer the population structure and to assign individual varieties to subpopulations. The models with a putative number of subpopulations (K) from 1 to 10 with admixture and correlated allele frequencies were considered [59]. Ten independent runs with 100,000 burn-in cycles and 100,000 iterations for each K were implemented based on trial runs of the program. Taking results from the STRUCTURE output file, the number of true clusters in the data (K) was determined using STRUCTURE HARVESTER [60], which identifies the optimal K based both on the posterior probability of the data for a given K and the ΔK [61]. The accessions and clones with membership probabilities ≥0.70 were considered to be of ‘pure’ ancestry versus membership probabilities ≤0.70 of ‘mixed’ ancestry.

## Results

### Transcriptome Sequencing and De Novo Assembly

**Figure 4:**
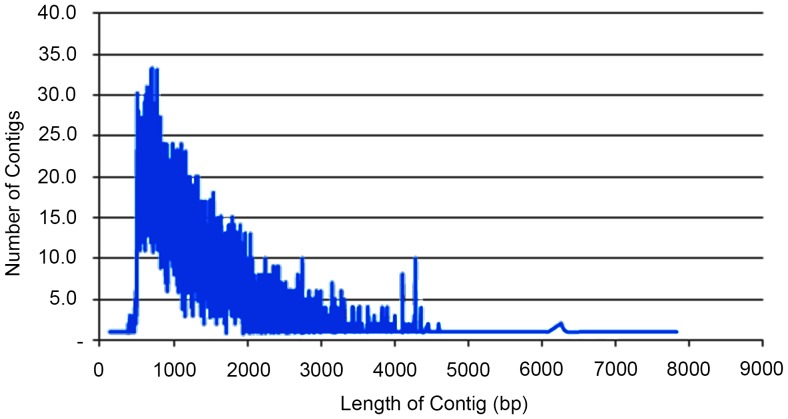
Length distribution of assembled Olive transcript contigs.

The olive genotypes Siyah Salamuralık, Yun Celebi, Yuvarlak Celebi, Hirhali Celebi and Halhali-3 were chosen among the Turkish olive genotypes as the most genetically diverse according to our genetic distance assessment based on the AFLP and SSR data. All sequencing processes were performed on an Illumina GAIIx. After transcriptome sequencing, a total of 159,978,483 raw reads were obtained from the five olive transcriptomes. The raw paired-end sequence data reported in FASTQ format was deposited in the National Centre for Biotechnology Information’s (NCBI) Short Read Archive (SRA) database under the accession number of NCBI:SRP026380. A summary of these sequencing results are presented in Table 3. After trimming for the adaptors and primer sequences, 18,504,103 sequences were removed due to their short length and 6,424,739 sequences were removed due to their low complexity and overall low-quality scores. Then, the ribosomal RNA sequences (8,507,228) were removed using the SILVA DB. The pre-assembly cleaning and trimming step resulted in 126,542,413 high-quality (HQ) reads, corresponding to 79% of the original raw sequences. A total of 126,542,413 high-quality cleaned reads ranging from 70 bp to 101 bp, with an average length of 97 bp, were harvested (Table 3). A total of 126,542,413 HQ reads were assembled using the Newbler software, which produced 22,052 contigs. The size of the contigs ranged from 136 to 7,827 bp, with an average length of 1321 bp. The size distribution of the contigs is shown in Figure 4. We obtained 22,052 contigs, of which 22,007 were isotigs and 45 were singletons. An overview of the sequencing statistics and assembly is outlined in Table 4 and Table 5 respectively.

**Table 3:** Summary of sequencing, trimming and removing RNA reads of the Illumina GAIIx reads of five Olive genotypes.

	RawReads	LowQualSeqTrimmedReads	Ribosomal RNARemovedReads	CleanupedReads
Siyah salamuralık	31,176,658	29,889,236	28,403,513	25,195,917
Yun çelebi	32,000,187	30,337,765	28,928,062	23,567,434
Yuvarlak çelebi	32,216,464	30,536,168	26,878,284	21,616,192
Hirhali çelebi	31,865,453	31,022,358	30,204,923	27,980,946
Halhali 3	32,719,721	31,768,217	30,631,734	28,181,924
Total	126,542,413			

**Table 4:** Statistic of olive transcriptome sequences.

Statistic of olive transcriptome sequencing	
Total number of raw reads	159,978,483
Total number of cleaned reads	126,542,413
Average length of cleaned reads	97 bp
Sequences for assembly	12,278,575,543 bp
Total number of short sequences removed	18,504,103
Total number of rRNA removed	8,507,228
Total number of low quality trimmed sequences	6,424,739

**Table 5:** Statistic of olive assembly.

Statistic of transcriptome assembly	
Total number of contigs	22,052
Minumum contig length	136 bp
Maximum contig length	7827 bp
Average contig length	1,321 bp
Total number of mapped reads	45,055,739
Total number of unmapped reads	81,486,674
Total number of singletons	45
Total number of isotigs	22,007
Total number of sequences for GO terms	105,570
Total Sequences at the molecular function level	22,644
Total sequences at the biological process level	55,523
Total sequences at the cellular component level	27,403
Total number of SNPs detected in transcriptomes	2,987

### Detection of Single Nucleotide Polymorphisms

The GATK Unified Genotyper Algorithm identified 2,987 high-quality putative cSNP primers in 22,052 contigs. The detailed information regarding the identified cSNPs is included in Table S1.

### Functional Classification by GO

**Figure 5:**
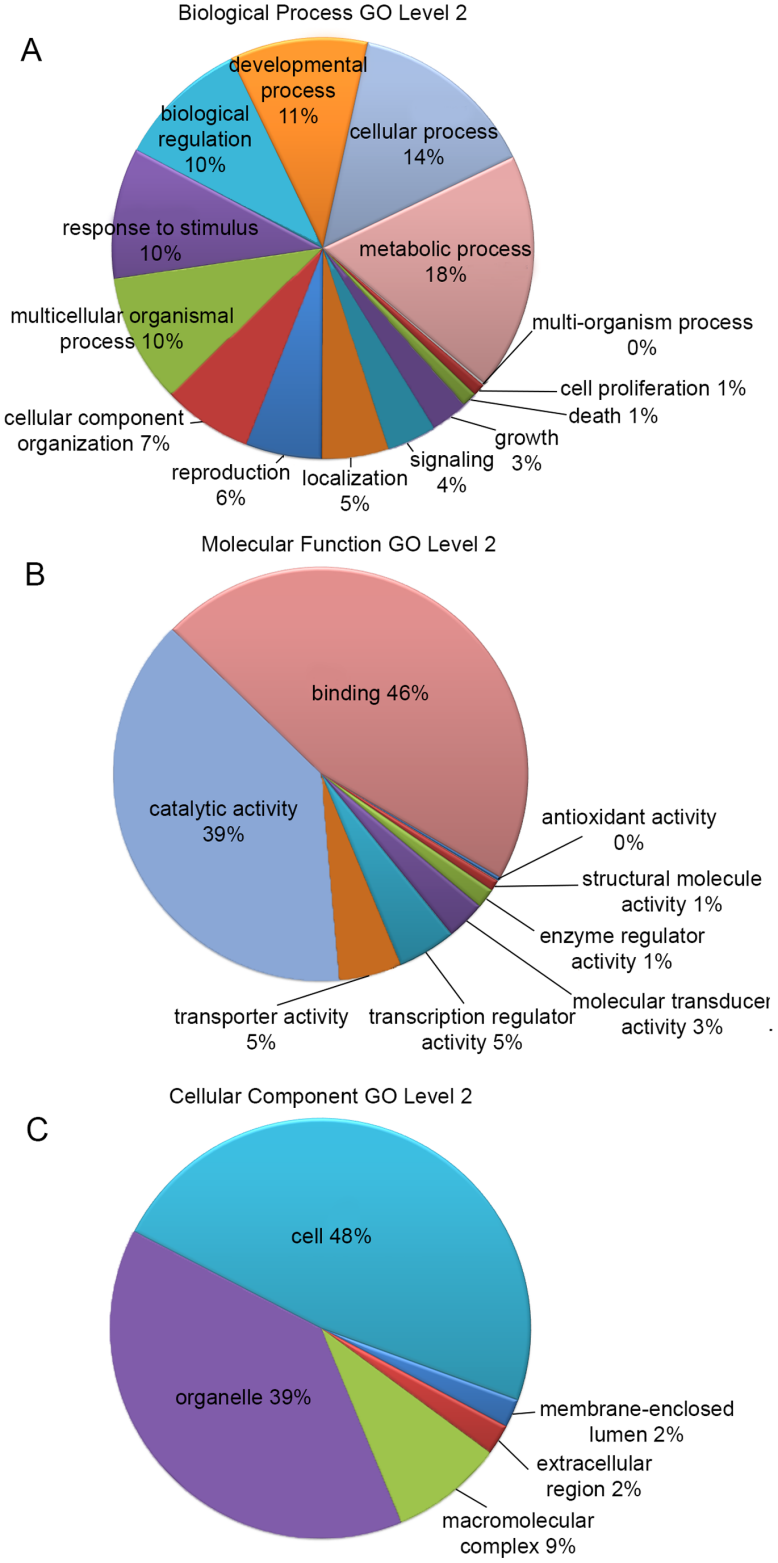
GO Classification. Olive transcriptome GO terms from level 2 of the biological process (A), molecular function (B) and cellular component (C) categories.

GO is an international standardized gene functional classification system and covers three domains: cellular components, molecular functions, and biological processes. To facilitate the organization of the olive transcripts into putative functional groups, GO terms were assigned using Blast2GO. A total of 105,570 sequences were assigned GO terms (Table 5), including 55,523 sequences at the biological process level, 22,644 sequences at the molecular function level, and 27,403 sequences at the cellular component level. The olive contigs were assigned based on GO terms using Blast2GO matches to align with a known function. The functional classification based on biological processes, molecular functions and cellular components is depicted in Figures 5A, 5B and 5C, respectively. Among the biological process terms, a significant percentage of genes were assigned to metabolic (18%) and cellular (14%) processes. Regarding molecular functions, a high percentage of sequences were assigned to binding (46%) and catalytic activity (39%), whereas many genes were assigned to cell parts (48%) and organelles (39%) for the cellular components functional class.

### Marker Polymorphism and Genetic Diversity

Twenty six AFLP primer combinations yielded 919 polymorphic bands. The number of polymorphic fragments ranged from nine (MCAA-EAGC) to 61 (MCAC-EACC), with an average of 35.3 fragments per primer combination (Table 6). A total of 62 alleles were obtained from the 14 SSR primer pairs. The number of alleles per locus ranged from two (GAPU82) to 8 (GAPU89 and GAPU103A), with an average of 4.4 alleles per locus (Table 6). AFLP and SSR profiles from representative gels are shown in Figure S1 and S2 respectively. Forty nine cSNP primers revealed 89 polymorphic amplified DNA fragments. The number of polymorphic fragments ranged from one to 8, with an average of 1.8 per primer combination (Table 2). The PIC values ranged from 0.01 to 0.99, with an average of 0.5 per fragment. To identify the power of resolution of the primers, the PIC values for all the polymorphic fragments generated by a primer were calculated to obtain an average PIC value for the corresponding primer combination. As a result, the highest PIC value (0.99) was observed for the primer SNP0089 and the lowest (0.01) was recorded for the primer SNP0017 (Table 2). All the microsatellite loci scored in this study were highly polymorphic, displaying high PIC values ranging from 0.49 (GAPU82) to 0.89 (GAPU90) (Table 6), with an average 0.69. The PIC values for the AFLP markers in the examined genotypes ranged from 0.22 (M-CAA/E-AAC) to 0.72 (M-CTA/E-AAG), with an average of 0.47 (Table 6).

**Table 6:** List of *Eco*RI+3/*Mse*I+3 AFLP primer combinations and SSR primers used with number of polymorphic fragments and PIC value.

AFLP Primer	A*	PIC	AFLP Primer	A*	PIC	SSR Primer	B*	PIC
M-CAA/E-AAC	21	0.22	M-CAC/E-ACT	59	0.44	ssrOeUA-DCA7	3	0.70
M-CAA/E-ACG	28	0.35	M-CAC/E-ACC	61	0.55	ssrOeUA-DCA11	7	0.74
M-CAA/E-ACA	50	0.52	M-CAC/E-ACG	57	0.56	ssrOeUA-DCA13	5	0.76
M-CAA/E-AGC	9	0.35	M-CAG/E-ACC	43	0.64	ssrOeUA-DCA15	4	0.62
M-CAA/E-AGG	24	0.54	M-CAG/E-AGG	43	0.59	ssrOeUA-DCA18	3	0.57
M-CAC/E-AAG	21	0.49	M-CAT/E-ACG	22	0.39	GAPU71A	3	0.83
M-CAC/E-AGC	26	0.60	M-CAT/E-AAG	42	0.24	GAPU71B	4	0.67
M-CAC/E-ACA	30	0.56	M-CAT/E-ACT	21	0.44	GAPU82	2	0.49
M-CAC/E-ACG	34	0.55	M-CAT/E-ACC	51	0.57	GAPU89	8	0.76
M-CAG/E-AAC	51	0.45	M-CTA/E-AAC	32	0.54	GAPU90	4	0.89
M-CAG/E-ACG	38	0.48	M-CTA/E-ACT	62	0.57	GAPU92	3	0.53
M-CAG/E-AAG	19	0.50	M-CTA/E-AAG	20	0.72	GAPU101	4	0.77
M-CAG/E-ACT	17	0.64	–	–	–	GAPU103A	8	0.81
M-CAC/EA-CA	38	0.53	–	–	–	GAPU108	4	0.84

A*: Number of polymorphic fragments obtained from AFLP primer combination.

B*: Number of alleles obtained from SSR primer.

The degrees of genetic similarity among the 96 olive genotypes based on AFLP, SSR and cSNP markers ranged from 0.24 to 0.75, with an average value of 0.49. The highest degree of intervarietal genetic diversity was found at the DNA level. The smallest degree of genetic similarity was 0.24 and was observed between “Yun celebi” (genotype 53) and “Melkabazı” (genotype 67). The two cultivars also differed greatly in their agromorphological characteristics. The maximum genetic similarity (0.75) was found between “Hurma Kaba” (genotype 72) and “Yag zeytini” (genotype 84). These two cultivars grow in western Turkey and exhibit very similar morphological characteristics.

### Hierarchical Cluster Analysis

**Figure 6:**
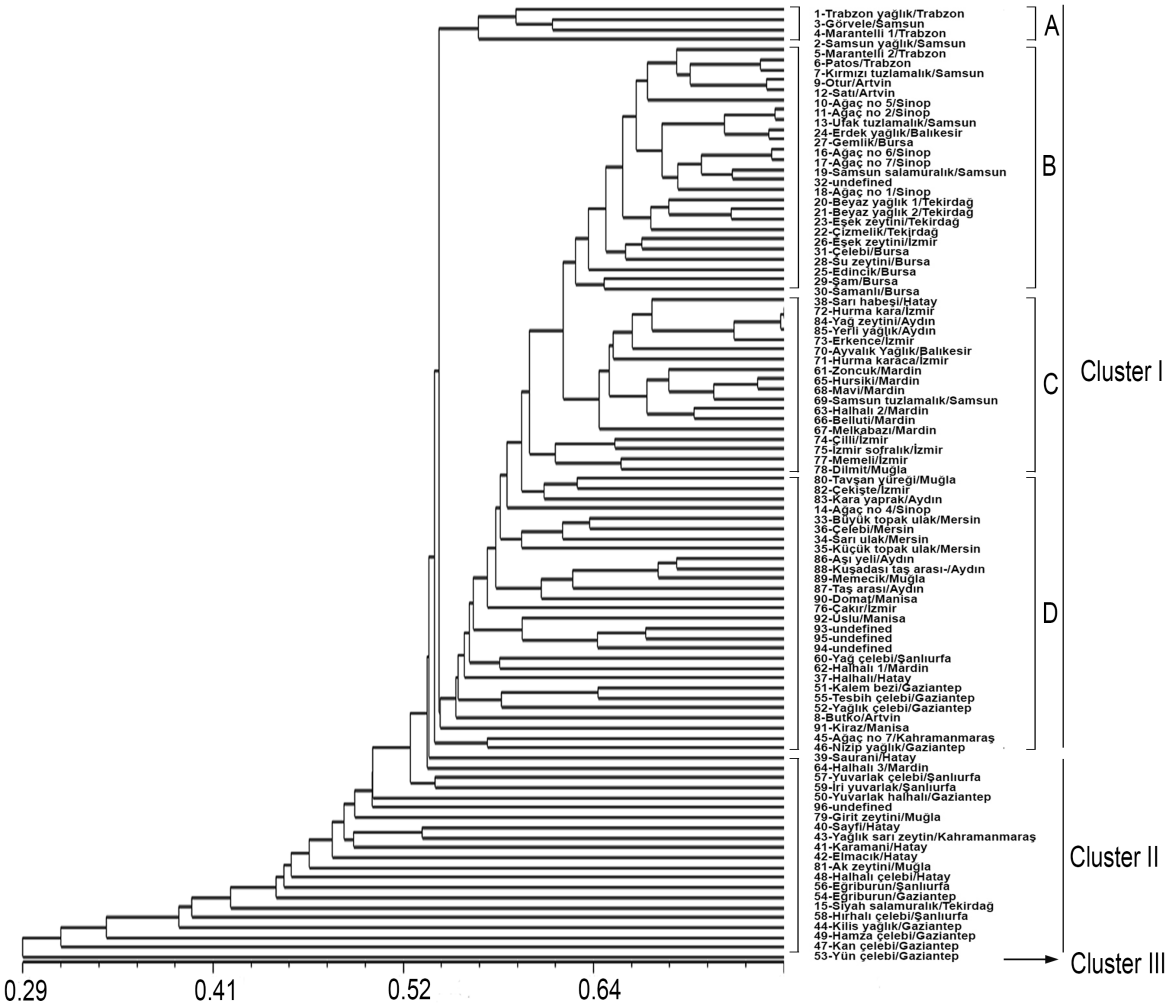
UPGMA dendrogram based on Jaccard’s coefficient illustrating the genetic similarities and distance among olive genotypes.

**Figure 7:**
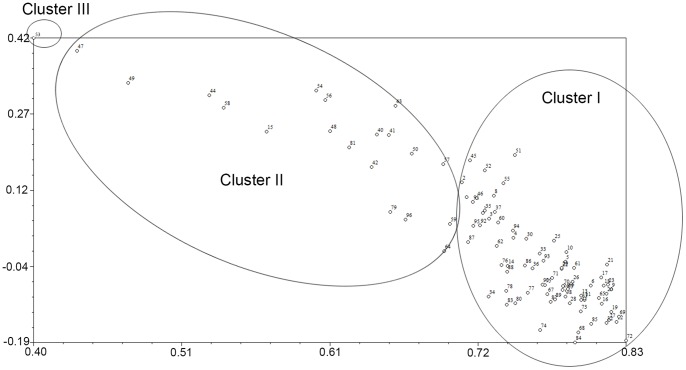
Principal coordinate analysis based on combined marker data showing distribution of 96 olive genotypes.

**Figure 8:**
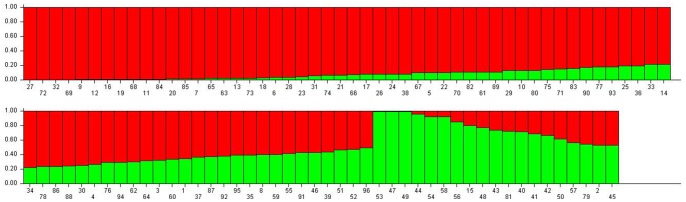
Bar plot diagrams for Structure. Codes are defined in Table 1. Each cultivar is represented by a vertical column, which is partitioned into K colored segments that represent the cultivar’s estimated common fractions in the K clusters.

The DNA marker-based genetic diversity among 96 olive genotypes was obtained using three different but complementary approaches, Neighbor Joining (NJ)-based hierarchical clustering (Figure 6), Principal Coordinate Analysis (PCA) (Figure 7), and Bayesian model-based clustering (Figure 8).

To obtain a more accurate clustering, a combined analysis was carried out using all of the AFLP, SSR and cSNP bands together. As shown in Figure 6, all of the examined genotypes could be classified into three major clusters using the UPGMA cluster analysis and the Jaccard similarity coefficients. This analysis clearly discriminated all of the cultivars, with genetic similarity coefficients between all possible pairs of genotypes ranging from 0.24 to 0.75. The largest number of genotypes included was in cluster 1, containing 75 genotypes that were subdivided into four sub-clusters (A, B, C, D) at a 0.56 genetic similarity. Sub-cluster A included five genotypes from the Black Sea Region at a 0.53 genetic similarity, and among them, ‘Gorvele” (genotype 3) and ‘Marantelli 1” (genotype 4) genotypes were the most similar (0.60). Group B contained 25 olive genotypes: twelve from the Black Sea Region, eleven from the Marmara Region, one from the Aegean Region and one undefined genotype that had a 0.58 genetic similarity. The highest genetic similarity value of 0.74 was observed between “Agac no 2” (genotype 11) and ‘Ufak tuzlamalık’ (genotype 13) in sub-cluster B. Sub-cluster C included 18 olive genotypes at a 0.56 genetic similarity that was composed of 9 genotypes from the Aegean Region, six from the Southeastern Region, and one each from the Mediterranean Region, the Marmara Region and the Black Sea Region. ‘Hurma Kaba’ (genotype 72) and ‘Yag zeytini’ (genotype 84) had the highest genetic similarity value (0.75) and were placed in sub-cluster C. The largest number of genotypes (28) occurred in sub-cluster D. These genotypes were more genetically distinct than those of the other sub-clusters, with an average genetic similarity of 0.55. Sub-cluster D is composed of eleven genotypes from the Aegean Region, six each from the Mediterranean Region and the Southeastern Region, two from the Black Sea Region and three undefined genotypes. Cluster II included 20 olive genotypes at 0.46 genetic similarity prevalently from the Southeastern Region. It is composed of 10 genotypes from the Southeastern Region, six from the Mediterranean Region, two from the Aegean Region, 1 from the Marmara Region and 1 undefined genotype. The most distant variety was ‘Yun celebi’ (genotype 53), which did not cluster with any other accessions. The genotype ‘Yun Celebi’ was not included in any of the groups, most likely because it has an independent origin.

A principal coordinate analysis separated the 96 olive genotypes into three major clusters, which was consistent with assignments generated by the UPGMA clustering analysis (Figure 7). The genotypes belonging to cluster I (as inferred by the UPGMA clustering analysis) were mainly distributed in the right portion of the resulting plot, with cluster II distributed in the upper right and cluster III in the upper left. The distribution of the genotypes of cluster I were more tightly clustered than those of cluster II, indicating that the genotypes in cluster II had a higher diversity than those of cluster I.

**Figure 9:**
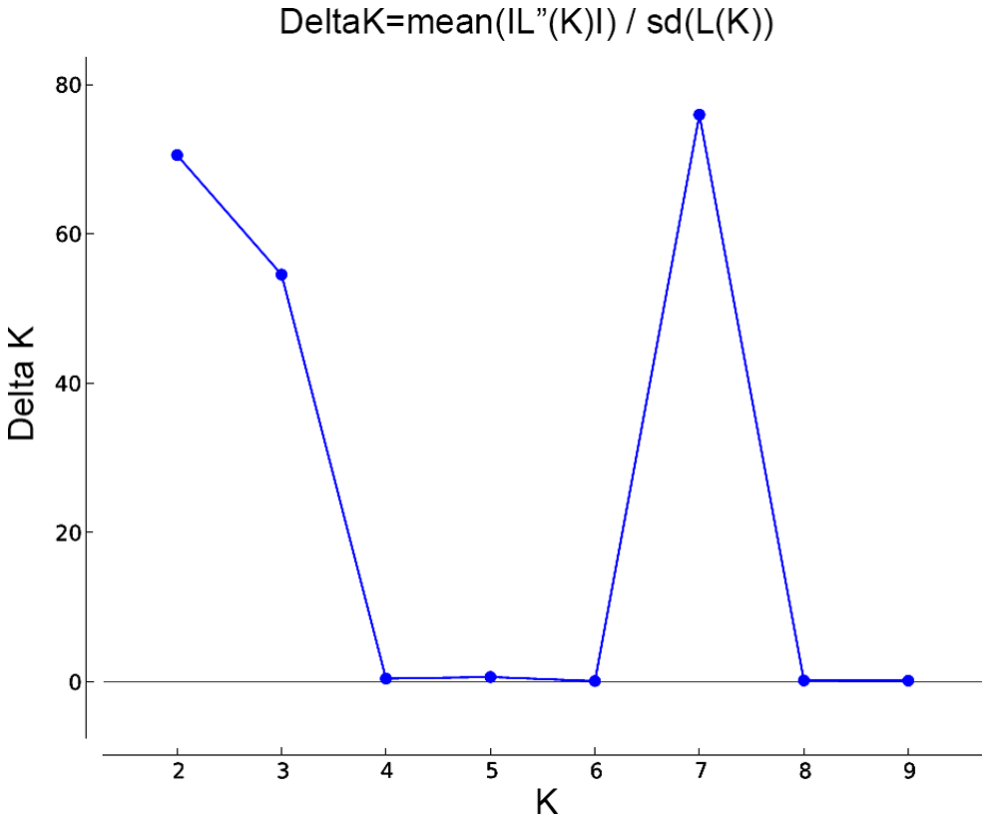
ΔK values over 10 runs for increasing K-values, from 2 to 9.

The number of subpopulations (K) was identified based on maximum likelihood and delta K (ΔK) values [61], to overcome the difficulty in interpreting the real K values. The probability of data increased at K = 2, and then steadily decreased up to K = 9 (Figure 9). The olive genotypes were separated into 2 populations (K = 2) based on the STRUCTURE analysis (Figure 8). The 70 assigned genotypes were structured into two groups, whereas the other 26 genotypes were retained in the admixed groups. The first inferred population, Group 1, consisted of 58 olive genotypes (fifteen from the Black Sea Region, eighteen from the Aegean Region, twelve from the Marmara Region, six from the Southeastern Region, four from the Mediterranean Region and 3 undefined genotypes). The second inferred population, Group 2, was comprised of 12 olive genotypes (five each from the Mediterranean and Southeastern Regions, one from the Marmara Region and one from Aegean Region). Twenty six genotypes (27.1%) showed membership values (q) lower than 0.70 and were categorized as admixture forms with varying levels of membership shared between the two clusters. Some members of the admixed genotype group showed a high level of admixture (p = 0.53). The distribution of the 96 genotypes that shared at least 70% ancestry with one of the two inferred groups is available for download as Table S2.

## Discussion

### Illumina Paired-end Sequencing, Assembly and SNP Marker Discovery

High throughput SNP discovery has been developed with the advent of NGS technologies, especially in those species lacking a reference genome [62]. NGS eliminates the expensive and time-consuming steps in traditional sequencing and permits the cost effective scoring of SNPs [10], [63]. In this study, to identify cSNPs from the transcriptome, NGS targeted only the coding DNA and ignored DNA from highly repetitive regions of the genome [64]. Illumina transcriptome sequencing has successfully been applied in model [65], [66] and non-model plant systems [67]–[69]. Until now, a limited number of SNP markers have been discovered for the olive. The current study was undertaken to discover cSNPs using five olive genotypes that were of diverse genetic backgrounds using Illumina GAIIx sequencing technology. The Illumina GAIIx platform was chosen to sequence the genotypes because of its high throughput, speed and relatively low cost [68], [70]. In the case of important tree species, transcriptome sequencing has been applied in eucalyptus [14], pine [71], black pepper [72] and various Prunus species [71], such as peach [73] and apricot [74]. In eucalyptus, Mizrachi et al. [14] developed an extensive expressed gene catalog for a commercially grown *E. grandis × E. urophylla* hybrid using Illumina mRNA-Seq technology. The Illumina runs generated 18,894 mRNA-derived contigs. Parchman et al. [71] described the 454 pyrosequencing of cDNA from Lodgepole pine (*P. contorta*) and assessed the utility of this approach for transcriptome characterization and marker discovery. The resulting 586,732 sequencing reads in the Parchman et al. [71] study have been assembled into 63,657 contigs and have identified 3,707 cSNP markers. Similar to our study, transcriptome sequencing using NGS technologies in tree species has been successfully implemented and generated high-quality reads for marker discovery. Compared with previous transcriptomic studies in other plants, such as Walnut [75], forest tree [76] and Jatropha curcas L. [77], we herein report additional contigs, suggesting that the olive contains very abundant gene resources. In accordance with the previous reports, our results also demonstrated that short reads from Illumina sequencing could be assembled and used in transcriptome analysis for cSNP marker development [70], [78]. In this study, approximately 13 million reads were generated from Illumina GAIIx and were finally assembled into 22,052 contigs, with an average length of 1,321 bp. This is the first study of a large scale transcriptome sequencing analysis of the olive in terms of sequencing reads (126,542,413) and the discovery of cSNPs. Alagna et al. [79] and Munoz-Merida et al. [80] obtained 261,485 and 1,932,337 reads, respectively, using 454 pyrosequencing. To understand the molecular basis of some important characteristics, such as fatty acid composition and phenolic and volatile compounds, Munoz-Merida et al. [80] applied Sanger and 454 pyrosequencing technologies to generate ESTs from different olive tissues and developmental stages. Similarly, Alagna et al. [79] performed transcriptome sequencing of cDNA from olive drupes to identify ESTs involved in phenolic and lipid metabolism during fruit development.

### Assessment of Marker Polymorphism and Genetic Diversity

This is the first study in which the genetic diversity, structure and characterization of 96 olive genotypes distributed over a large area in Turkey were compared using cSNP, AFLP and SSR markers. Each genotype analyzed has agronomical and economic importance for the olive oil or table olive industries. Several previous studies regarding olive genetic diversity applied molecular marker systems that aimed to acquire a unique and comprehensive genetic cultivar characterization in the most important olive collections, such as the World Olive Germplasm Bank of Cordoba, Spain [81]–[84], the Germplasm Collection of Valencia, Spain [85], the CBNMP Olive Collection, France [86], the germplasm collection of the United States Department of Agriculture in Davis, USA [87], the Olive Collection in Israel [88], and the Olive Collection Orchard in Slovenia [89]. In this study, we have attempted to characterize the Turkish olive genotypes in the Center for Turkish Olive GenBank Resource (CTOGR) using AFLP, SSR and cSNP markers to describe, to conserve their germplasm and to identify individuals who would represent suitable parents for a breeding program aimed at enhancing the quality of olive oil and table olives. The analyses of our marker type data revealed that the Turkish genotypes contain substantial diversity, which could support the national breeding program’s objectives as well as allow for the participation in international programs aiming at olive improvement and conservation.

In the past, a variety of molecular markers such as, ISSRs, SSRs, SRAPs, DARTs and AFLPs have been used to estimate the genetic diversity in olive genotypes of different origins [29], [32]. Recently, a limited number of SNP markers (10) developed from some gene regions have also been used for the estimation of genetic diversity in the olive [32], [42], [43]. The use of a particular molecular marker type to estimate the genetic diversity of germplasm collections, however, depends on many factors, including the cost to genotype a large population with a marker assay [29]. In recent years, the SSR and SNP markers, due to their inexpensive developmental costs [29], have increasingly being used for the genotyping of natural or breeding populations. Together with these markers, AFLP markers are still considered good for fingerprinting or diversity analyses [30], [90]. Therefore, the present study documents the combined utility of these marker types for genetic diversity studies.

All of the AFLP and SSR markers used in this study showed a high level of polymorphism in all of the olive genotypes examined in the present and previous studies [52], [53], [91]. In this study, 26 AFLP primer combinations yielded 919 polymorphic bands. The number of polymorphic bands per primer pair ranged from 9 (MCAA-EAGC) to 61 (MCAC/EACC), with an average of 35.3. Angiolillo et al. [91] and Baldoni et al. [92] obtained similar results regarding the number of bands per primer pair. Taamali et al. [93] also obtained similar results with their 74 fragments per primer combination. However, the number of alleles per SSR primer in our study was lower than Taamali et al. [93]. In the present study, 62 alleles were detected from 14 SSR primers. The obtained numbers of alleles generally agree with recent olive SSR studies [52], [83], [94]–[97]. According to previous research [37] carried out in a sample of 561 accessions from 14 Mediterranean countries, very high genetic variation has been detected using SSR primers. A high variability of microsatellites in the olive was also shown by Belaj et al. [95], whose analyses included 35 Spanish and Italian olive varieties assayed with nine SSR markers, giving an average number of 7.5 alleles per locus. Lopes et al. [94] also obtained similar results: 9.6 alleles on average over 14 microsatellites loci in 130 olive samples originating from different areas in Europe. These results may be due to the large collection of diverse samples, increasing the chance of obtaining polymorphic SSR alleles. On the other hand, Isik et al. [36] reported 89 alleles from 13 SSR primers in the Turkish olive varieties, with an average of 6.8 alleles per primer. They included three European olive varieties from Spain, Italy and France as outgroups. As already evidenced in previous studies [98], AFLP, SSR and cSNP analyses may result in differences in the absolute estimates of genetic variation and divergent results according to the olive genotypes used.

The SSR and cSNP markers were highly polymorphic, while the AFLP markers showed a lower level of polymorphism for the germplasm examined in the present study. The high level of polymorphism associated with SSRs is expected due to the unique mechanism responsible for generating SSR allelic diversity [52], [53]. The two methods, in fact, amplify different types of genomic regions, and while AFLPs are designed to randomly sample regions from the whole genome, SSR markers specifically detect pre-identified repeat regions [99].

To evaluate the informativeness and efficiency of AFLP, SSR and cSNP markers in the analysis of genetic diversity as well as the population differentiation assessments, the PIC values for each marker locus were estimated. The PIC values for all primers ranged from 0.01 (SNP0017) to 0.99 (SNP0089). The PIC values for the twenty six AFLP primer combinations ranged from 0.21 (MCAA/EAAC) to 0.72 (MCTA/EAAG), with an average of 0.50. The average PIC values for the fourteen SSR loci was 0.71, and among the 96 olive cultivars, the PIC values ranged from 0.49 for GAPU82 loci and 0.88 for GAPU90 loci. The three SSR loci with PIC values above 0.80, GAPU71A, GAPU103A and GAPU108, showed total allele numbers of 3, 8 and 4, respectively. These numbers indicate that markers with a large number of alleles are informative for population studies. Bandelj et al. [89] reported similar PIC values with the same primer pairs in olive cultivars using SSR markers. Slightly higher PIC values (0.47 to 0.91) were registered by Belaj et al. [31] between local cultivars and wild olive trees from 3 important Spanish olive-growing regions. Do Val et al. [100] reported that the informativeness of the 12 loci (PIC) were highly variable (0.12–0.72), of which eight loci showed PIC values ≥0.50. These results are partially similar to ours.

Genetic diversity is an important index representing the genetic variation in olive genotypes. The high level of genetic diversity implies abundant germplasm variation, allowing for the selection of more useful genes for management and breeding programs. In this study, the genetic similarity coefficients ranged from 0.24 to 0.75. The results indicated that the 96 olive genotypes possessed a high-level of genetic variation. The genetic similarity values found in our study are comparable to the values previously found in other studies for the identification of Turkish olive genotypes using the AFLP [101], SSR [36], [44], and SRAP [36] markers. Isık et al. [36] investigated the genetic diversity in 66 Turkish olive varieties using SSR markers. The genetic similarity coefficients ranged from 0.45 to 0.90, with a mean of 0.68, indicating good variability among the genotypes studied [36]. In the present study, maximum genetic similarity (0.75) was found between ‘Hurma kaba’ (genotype 72) and ‘Yag zeytini’ (genotype 84). Similarly, ‘Yag zeytini’ and ‘Yerli yaglik’, at a 0.75 genetic similarity, are placed together in the same sub-cluster (sub-cluster C). Similar to our study, Isık et al. [36] showed 3 pairs of varieties (Celebi and Halhali, Hurma kaba and Yerli Yaglik, and Asi Yeli and Memecik) as having a genetic similarity index of nearly 0.90. In our study, the genetic similarity between ‘Çelebi’ (genotype 31) - ‘Halhalı’ (genotype 37) and ‘Aşı yeli’ (genotype 86) - ‘Memecik’ (genotype 89) were 0.57 and 0.68, respectively. These genotypes were placed in the same sub-cluster (D). Compared to our results, the greater genetic similarity indexes between these genotypes reported by Isık et al. [36] could be due to utilizing only SSR markers. However, in this study, the differences between genotypes were examined with more precision by utilizing 3 different marker techniques (AFLP, SSR and cSNP) that represent different loci. The use of different markers in combination to assess the genetic diversity is more precise in terms of reliability compared to the use of only one marker type [32], [102].

### Hierarchical Cluster Analysis

A phylogenetic tree constructed using the UPGMA clustering analysis revealed three major groups of olive genotypes that were congruent with geographical distribution patterns. In the olive, based on AFLP technology, Angiolillo et al. [91] showed that wild olive and cultivars from the Western Mediterranean Region did not cluster together, and they were relatively distant. However, a few oleasters clustered with the cultivars. Grati-Kamoun et al. [103] found that oil and table olive cultivars originating from Tunisia, based on AFLP, did not show any evidence of clustering according to their geographic origin. These two studies showed that the genotypes used might not correspond to their origin. However, in the present study, the genotypes used partially represent their origin and the genetic variation is very high. Similar to our results, Ercisli et al. [101] and Isik et al. [36] also showed that a high level of genetic variation exists in the Turkish olive germplasms. As in other olive-growing countries, the use of homonyms and synonyms in the designation of genotypes is a problem in the Turkish olive genotypes. The synonyms and homonyms among fruit trees have been widely reported [104]–[108]. Several previous studies noted several synonyms and homonyms in the olive collection, making it difficult to identify the reference of olive cultivars [29], [36], [53], [96], [100], [109]–[112]. Our results indicated that a number of accessions known by the same names were genetically different, suggesting that these were homonyms. Many genotypes, such as the two ‘Esek zeytini’ (genotype 23 and 26), the two ‘Çelebi’ (genotype 31 and 36), the two ‘Egriburun’ (genotype 54 and 56), and the two ‘Tas arasi’ (genotype 87–88), have similar names but different origins and did not cluster in the dendrogram and seemed to be homonymous. According to the genetic similarity matrix, the two ‘Esek zeytini’ (genotypes 23 and 26) and the two ‘Celebi’ (31 and 36) genotypes had genetic similarity values of 0.66 and 0.63, respectively. The two ‘Egriburun’ (genotype 54 and 56) genotypes had a genetic similarity value of 0.43, while the two ‘Tas arasi’ (genotype 87–88) genotypes had a 0.65 genetic similarity. The presence of homonyms in olive genotypes has been previously reported by Ozkaya et al. [113] and Isik et al. [36]. Several genotypes of the ‘Derik Halhali’ olive were found to be molecularly and morphologically different [113]. Similar to our study, ‘Egriburun’, ‘Celebi’, and ‘Tas arasi’ genotypes were identified as homonyms [36].

The model-based STRUCTURE analysis used herein revealed the presence of two groups among the collected genotypes. The grouping patterns obtained from the genetic similarity matrix and model-based membership differed somewhat (Figure 5 and Figure 6). For example, some genotypes from Group 1 of the STRUCTURE analysis were placed in Clusters 2 and 3 of the NTSYSpc-based dendrogram. These results were confirmed by Bayesian analyses, demonstrating that the olive genotypes have a complex genetic structure. This indicates the presence of a highly heterozygous genome in the Turkish olives. The distribution of the 70 genotypes that shared >70% ancestry with one of the two inferred groups is summarized in Table S2. Another 27.1% of the genotypes showed evidence of mixed ancestry, but the groupings were mostly inconsistent with the patterns of origin according to the STRUCTURE results. Studies on genetic structure have also been conducted on wild olive trees (or oleasters) and have been used to investigate the genetic relationships between wild and cultivated olives [32], [87], [92], [114]. Baldoni et al. [92] used AFLP markers to examine the genetic structure of wild and cultivated olives in the Central Mediterranean Basin, and the observed patterns of genetic variation were able to distinguish wild from cultivated populations and continental from insular regions. The genetic structure among the germplasm collections consisting of hundreds of olive cultivars was recently characterized using SSRs [87]. However, the different methods of cluster analysis used in these studies (e.g., hierarchical clustering, PCA analysis and STRUCTURE analysis) failed to distinguish between the olive cultivars of different origins. Sarri et al. [114] conducted a study of the genetic relationships based on SSRs among 118 cultivars sampled in several Mediterranean countries and showed that the Mediterranean olive germplasm was structured into three main gene pools, corresponding to the western, central and eastern Mediterranean Regions. Belaj et al. [32] reported that the STRUCTURE and PCA analyses revealed a certain clustering of the majority of olive accessions according to their regional origin, with the accessions from the eastern and western Mediterranean being the most differentiated.

## Conclusions

This study provides the first comprehensive transcriptome sequencing data used for the cSNP discovery of olive genotypes. We generated 126 million paired-end reads comprising 22,052 contigs from five distant genotypes, and 2.987 cSNP primers were identified. Our results demonstrate that high-throughput transcriptome sequencing is an efficient and effective way to identify a large numbers of cSNPs. The developed cSNP markers could aid in future studies of population genetics, QTL, association mapping studies and marker-assisted breeding in the olive. Further, based on the transcriptome analysis, new specific sequences could be used to design microarray chips, detection probes or PCR primers for different olive genotypes. In this study, 96 olive genotypes originating from different regions of Turkey were identified using these cSNPs in combination of AFLP and SSR DNA markers. The present results support the earlier suggestions that AFLP and SSR markers provide good insight into the genetic diversity of 96 olive genotypes. The information regarding the large-scale genetic diversity among Turkish olive genotypes could be used for their proper identification and for improving olive quality by breeding programs.

## Supporting Information

Figure S1 AFLP profiles showing the genetic polymorphisms among 96 olive genotypes using the selective primer combination of ‘M-CAA/E-AGG’. The figure displays the code numbers (as shown in Table 1) 1–48 (A) and 49–96 (B). “M” indicates the IRDye labeled 50–700 bp fragment size ladder (LI-COR, USA).
(TIF)

Figure S2 SSR profiles showing the genetic polymorphisms among 96 olive genotypes using the primer DCA13. The figure displays the code numbers (as shown in Table 1) 1–48 (A) and 49–96 (B). “M”indicates the IRDye labeled 50–700 bp fragment size ladder (LI-COR, USA).
(TIF)

Table S1 Sequence information of all of the cSNP primer pairs identified and designed using the GATK Unified Genotyper Algorithm. This file contains all of the information (sequence information, sequence length, and forward and reverse primer sequences) of the cSNP primer pairs designed using the GATK Unified Genotyper Algorithm.
(XLSX)

Table S2 The distribution of the 96 genotypes that shared at least 70% ancestry with one of the two inferred groups.
(XLSX)
