# Supplementary figures and images for: SNP Discovery by Illumina-Based Transcriptome Sequencing of the Olive and the Genetic Characterization of Turkish Olive Genotypes Revealed by AFLP, SSR and SNP Markers
Source: PLoS One. 2013 Sep 13;8(9):e73674. doi: 10.1371/journal.pone.0073674 (PMC3772808; doi:10.1371/journal.pone.0073674)

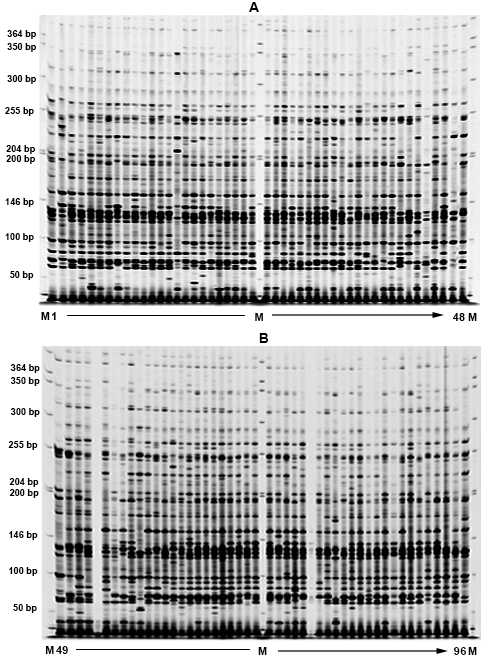

Supplement: Figure S1 — AFLP profiles showing the genetic polymorphisms among 96 olive genotypes using the selective primer combination of ‘M-CAA/E-AGG’. The figure displays the code numbers (as shown in Table 1) 1–48 (A) and 49–96 (B). “M” indicates the IRDye labeled 50–700 bp fragment size ladder (LI-COR, USA). (TIF) [file pone.0073674.s001.tif]

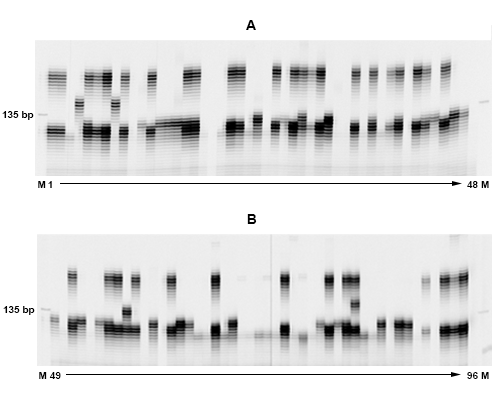

Supplement: Figure S2 — SSR profiles showing the genetic polymorphisms among 96 olive genotypes using the primer DCA13. The figure displays the code numbers (as shown in Table 1) 1–48 (A) and 49–96 (B). “M”indicates the IRDye labeled 50–700 bp fragment size ladder (LI-COR, USA). (TIF) [file pone.0073674.s002.tif]
